# Identification of muscle synergies associated with gait transition in humans

**DOI:** 10.3389/fnhum.2015.00048

**Published:** 2015-02-10

**Authors:** Shota Hagio, Mizuho Fukuda, Motoki Kouzaki

**Affiliations:** ^1^Research Fellow of the Japan Society for the Promotion of ScienceTokyo, Japan; ^2^Laboratory of Neurophysiology, Graduate School of Human and Environmental Studies, Kyoto UniversityKyoto, Japan; ^3^Faculty of Integrated Human Studies, Kyoto UniversityKyoto, Japan

**Keywords:** muscle activity, walking, running, motor control, electromyogram, non-negative matrix factorization

## Abstract

There is no theoretical or empirical evidence to suggest how the central nervous system (CNS) controls a variety of muscles associated with gait transition between walking and running. Here, we examined the motor control during a gait transition based on muscle synergies, which modularly organize functionally similar muscles. To this end, the subjects walked or ran on a treadmill and performed a gait transition spontaneously as the treadmill speed increased or decreased (a changing speed condition) or voluntarily following an experimenter’s instruction at constant treadmill speed (a constant speed condition). Surface electromyograms (EMGs) were recorded from 11 lower limb muscles bilaterally. We then extracted the muscle weightings of synergies and their activation coefficients from the EMG data using non-negative matrix factorization. As a result, the gait transition was controlled by approximately 9 muscle synergies, which were common during a walking and running, and their activation profiles were changed before and after a gait transition. Near a gait transition, the peak activation phases of the synergies, which were composed of plantar flexor muscles, were shifted to an earlier phase at the walk-to-run transition, and *vice versa*. The shifts were gradual in the changing speed condition, but an abrupt change was observed in the constant speed condition. These results suggest that the CNS low-dimensionally regulate the activation profiles of the specific synergies based on afferent information (spontaneous gait transition) or by changing only the descending neural input to the muscle synergies (voluntary gait transition) to achieve a gait transition.

## Introduction

In daily life, humans intuitively select their appropriate gait pattern, either walking or running, depending on their gait speed. As their gait speed increases, people shift their mode of locomotion from walking to running at a characteristic speed or *vice versa* (Thorstensson and Roberthson, 1987). Thus, people perform gait transitions: walk-to-run and run-to-walk. In this study, we sought to identify a neural interpretation for the human gait transition between walking and running, i.e., how the central nervous system (CNS) controls gait transition.

The idea that a gait transition (which occurs when the gait speed increases) is triggered by metabolic energy expenditure was initially proposed on the basis that humans adjust their walking and running gaits to minimize the metabolic energy cost of locomotion (Cavagna and Franzetti, 1986; Mercier et al., 1994; McNeill Alexander, 2002). The metabolic hypothesis, however, cannot completely explain the subsequent studies noting that the transitions from trotting to galloping in horses (Farley and Taylor, 1991) and from walking to running in humans (Hreljac, 1993a; Minetti et al., 1994; Brisswalter and Mottet, 1996) occur at speeds slower than do those predicted by the metabolic cost hypothesis. Farley and Taylor (1991) proposed that the trot–gallop transition reduces the peak forces of the muscles but that the walk–run transition in humans cannot be accounted for in the same way because the transition from walking to running increases peak vertical ground reaction forces (Nilsson and Thorstensson, 1989; Hreljac, 1993b). Additional observations supported other mechanical factors in humans as the trigger of a gait transition: the angle between the thighs (Minetti et al., 1994) and peak ankle angular velocity (Hreljac, 1995) abruptly decrease at the walk-to-run gait transition. However, these mechanical variables cannot explain the run-to-walk gait transition as gait speeds decrease (Kram et al., 1997). The trigger of a gait transition was also examined in the neural approach measuring electromyogram (EMG), which showed the different trigger muscles between walk-to-run and run-to-walk transitions (Prilutsky and Gregor, 2001) or the change of the preferred walk-to-run transition speed due to the changing of the demand on trigger muscles (Bartlett and Kram, 2008). In either case, however, almost all of the studies have focused on why a gait transition occurred. The problem remains how the CNS control the muscles associated with a gait transition.

Locomotion requires dynamic and precise coordination of multiple trunk and limb muscles via hierarchical neural pathways. To simplify redundant motor control, the CNS may control motion through muscle synergies, which modularly organize functionally similar muscle groups that are combined fundamentally depending on the purpose of the task (Tresch et al., 1999; Ting and Macpherson, 2005; Roh et al., 2012; Hagio and Kouzaki, 2014). During walking, specific muscle synergies are recruited, and the order of recruitment is consistent across patterns at particular time points of the gait cycle (Clark et al., 2010; Chvatal and Ting, 2013). Other researchers have reported that the activation pattern in walking is coordinated by five temporal modules (Ivanenko et al., 2004), which are the same across different walking speeds and during running (Cappellini et al., 2006). That study then concluded that the activation timing of one temporal module was distinctly different between walking and running (Cappellini et al., 2006). However, how the temporal module was changed during a gait transition is not clear. Hence, it is necessary to clarify the modular control with muscle synergies in a continuous change of gait pattern.

Therefore, the main purpose of this study was to examine motor control during the gait transition between walking and running based on muscle synergies. We examined two different types of gait transition: (1) a spontaneously transition as gait speed gradually changed; and (2) a voluntarily transition regardless of gait speed. To clarify the two different controlling strategies, subjects walked or ran on a treadmill, and we observed the gait transition between the gait patterns (the walk-to-run and run-to-walk transitions) using two different approaches: (1) constantly accelerating or decelerating the treadmill; and (2) following the instructions of an experimenter with the treadmill speed held constant. During these tasks, we recorded muscle activity and extracted muscle synergies using a decomposing technique. Our successive results demonstrated neural mechanisms during the gait transition between walking and running.

## Methods

### Subjects

Five healthy male subjects (age = 24.3 ± 1.6 yr., height = 170.8 ± 4.7 cm, weight = 65 ± 5.6 kg, mean ± SD) participated in this study. All subjects gave their written informed consent prior to the experiment. The experimental procedures were conducted in accordance with the Declaration of Helsinki and were approved by the Local Ethics Committee of the Graduate School of Human and Environmental Studies, Kyoto University (25-H-40).

### Experimental protocol

The experiments were carried out on a treadmill (Adventure 3 PLUS, Horizon, Johnson Health Tech Japan Co., Tokyo, Japan). The walking surface of the treadmill was 1.41 m long and 0.5 m wide. The subjects walked or ran on the treadmill. Prior to the trials, all subjects were familiarized with treadmill walking and running by performing treadmill locomotion at 4.0 km/h (walking) and 8.0 km/h (running) for ~5 min across each speed. In this study, two different conditions were conducted: (1) a changing speed condition; and (2) a constant speed condition. During the changing speed condition, the subjects were asked to perform their own gait pattern, either walking or running, and included a natural gait transition depending on the treadmill speed (set to constantly accelerate or decelerate) (Segers et al., 2006). The subjects started walking at 3.0 km/h, and the treadmill speed was continuously increased 0.1 km/h every 1 s. After the walk-to-run transition, the subjects ran for 20 s with constant speed, which was adequately faster than the walk-to-run transition speed. The speed was then decreased to 3.0 km/h, and the run-to-walk transition occurred in a similar fashion. Each subject performed five successful trials (30 s rest between each trial). In the constant speed condition, the subjects were asked to walk or run for 10 s in turn at the walk-to-run transition speed, which was determined as the average transition speed for the 5 changing speed trials, following an experimenter’s instruction. One trial period was approximately 100 s, and each gait pattern was repeated 5 times. The trials were repeated until three successful trials were recorded (30 s rest between each trial). For all trials, an experimenter controlled the speed of the treadmill, and the treadmill controller panel was not visible to the subject.

### Experimental setup

Surface EMGs were recorded from 11 muscles spanning the ankle, knee and hip joint bilaterally (22 muscles). The electrode placement was carefully chosen to minimize crosstalk from the adjacent muscles. The recorded muscles were soleus (SOL), medial gastrocnemius (MG), lateral gastrocnemius (LG), tibialis anterior (TA), rectus femoris (RF), vastus lateralis (VL), vastus medialis (VM), biceps femoris long head (BFL), biceps femoris short head (BFS), gluteus medius (GMed) and gluteus maximus (GMax). The EMGs were recorded using bipolar Ag-AgCl electrodes. Each electrode had a diameter of 5 mm, and the inter-electrode distance was 10 mm. A small inter-electrode distance was used to prevent crosstalk among neighboring muscles (Imagawa et al., 2013). A reference electrode was placed on the lateral epicondyle of the femur. The EMG signals were amplified (MEG-6116M, Nihon-kohden, Tokyo, Japan) with band-pass filtering between 5 and 1000 Hz. All electrical signals were stored with a sampling frequency of 2000 Hz on the hard disk of a personal computer using a 16-bit analog-to-digital converter (PowerLab/16SP; AD Instruments, Sydney, Australia). The raw EMG traces were high-pass filtered at 100 Hz using a zero-phase-lag fourth-order Butterworth filter and were demeaned, digitally rectified and low-pass filtered at 15 Hz (Ivanenko et al., 2004).

In this study, we determined the gait steps using footswitches attached to both the toes and heels within the shoes (Wall and Crosbie, 1996). One gait cycle was defined as right heel contact to the moment before the next right heel contact. Walking and running were generally determined by whether a double stance phase was present or absent. Walking has a double stance phase, and running is characterized by a flight phase (Whitall and Caldwell, 1992; Getchell and Whitall, 2004; Titianova et al., 2004). Hence, we judged walking and running by whether the left toe was in contact (or not) when the right heel was contact. The transition step was defined as the first step with a flight phase (the walk-to-run transition) or the first step with a double stance phase (the run-to-walk transition) and was called step zero (0; Thorstensson and Roberthson, 1987; Li, 2000; Li and Hamill, 2002; Segers et al., 2006).

### Data processing

To identify muscle synergies, we first generated an EMG data matrix across each subject. The EMG traces were time-interpolated over individual gait cycles from the onset of the right leg to the next to fit a normalized 200-point time base. We analyzed the data from 8 steps before to 8 steps after the transition (total 17 cycles) to identify the possible occurrence of a transition process that facilitates the actual realization of transition. In the constant speed condition, the steps before and after each six successive gait transitions, including three walk-to-run transitions and three run-to-walk transitions, were used for analysis. In the extraction of muscle synergies, we assumed that people control both walking and running through the same muscle synergies based on the previous evidence (Cappellini et al., 2006) and our verification of the idea (see below). Hence, we extracted muscle synergies from the total EMG matrix, which included walking, running and gait transition steps. Furthermore, because recent study provided evidence that the activation patterns of temporal modules during walking were bilaterally linked (Maclellan et al., 2014), we also assumed that muscles in both legs were modularly connected with muscle synergies. Thus, we generated an EMG data matrix, which consisted of 17 cycles × 200 time periods × 20 repetitions (5 repetitions and 15 repetitions; the changing and constant speed conditions, respectively) = 68000 time bins for each of the 22 muscles in both sides. The EMG data matrices were normalized to their respective maximum amplitude so that all muscle scales ranged from 0 to 1. Prior to extracting muscle synergies, each muscle vector in the data matrix was normalized to have unit variance and thus ensure that the activity in all muscles was equally weighed.

### Extraction of muscle synergies

We extracted muscle synergies from each EMG data matrix using non-negative matrix factorization, “NMF” (Lee and Seung, 1999; Tresch et al., 1999; Ting and Macpherson, 2005; Torres-Oviedo and Ting, 2007; Hagio and Kouzaki, 2014). NMF assumes that a muscle activation pattern *M*, in a given time period is composed of a linear combination of a few muscle synergies, *W_i_*, that are each recruited by a synergy recruitment coefficient *C_i_*. Therefore, a particular muscle activation pattern* M*, would be represented by

$$M=\sum_{i=1}^{N}W_{i}C_{i}+\varepsilon \left(W_{i}\geq 0,C_{i}\geq 0\right)$$

where we specify the relative contributions of the muscles involved in synergy, *i*. Each muscle synergy has a fixed composition, *W_i_* and is multiplied by a scalar recruitment coefficient, *C_i_*, which changes over time and across conditions. *ε* is residual. The synergy weighting and activation coefficient matrices were normalized such that the individual weighting vector was the unit vector (Hagio and Kouzaki, 2014).

To select the number of muscle synergies that could best reconstruct our data, we extracted between 1 and 22 synergy matrices and synergy activation coefficient matrices from the EMG data matrices that were obtained from each subject. Then, we verified the goodness-of-fit between the original (EMG_o_) and reconstructed (EMG_r_) data matrices; the data matrices were calculated using NMF analysis to select the smallest number of muscle synergies (N_syn_) that resulted in an adequate reconstruction of the muscle responses. We first calculated the variability accounted for (VAF) as 100 × the coefficient of determination from the uncentered Pearson correlation coefficient (Zajac, 1989; Torres-Oviedo et al., 2006), which was based on the entire dataset (global VAF). VAF is sensitive to both the shape and the magnitude of the original and reconstructed datasets. The number of muscle synergies underlying each dataset was defined as the minimum number of synergies required to achieve a global VAF > 95% and a mean VAF for each muscle (muscle VAF) that exceeded 80%. For N_syn_ muscle synergies, both a synergy weighting and synergy activation coefficient matrix were defined.

### Verification of the analysis

**Figure 1:**
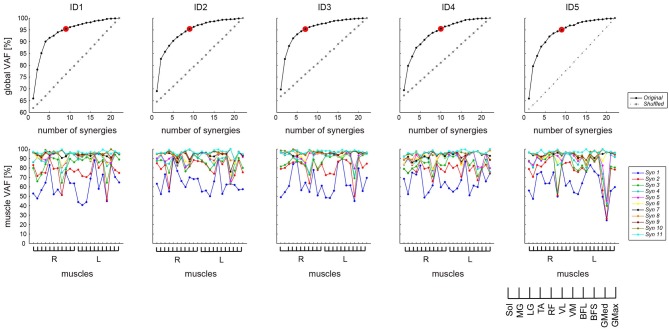
**VAF to determine the number of synergies**. *Top*: The global VAF as a function of the number of synergies used for reconstruction was based on the original (black solid line) and shuffled (gray dashed line) EMG datasets. In all cases, the VAF values for the reconstruction of the original data using the identified number of synergies (indicated by red circle) were higher than the VAF values for the shuffled datasets. *Bottom*: The VAF across 11 muscles, bilaterally. Different lines indicate the different numbers of muscle synergies. VAF, variability accounted for. Syn, synergy.

To verify that the extracted muscle synergies were due to the inherent organization of muscle activation based on neurophysiological evidence rather than artifacts of the NMF method, the VAF levels for the synergies extracted from the original data were compared with the VAF values for synergies extracted from shuffled datasets. For the shuffled procedure, the data for each muscle were shuffled independently (Chvatal et al., 2011). Figure 1 (top) shows the plots of both the original (black solid line) and shuffled (gray dashed line) global VAF values in the condition extracting muscle synergies as a function of the number of synergies across subjects. This comparison showed that the VAF values with the appropriate number of synergies extracted from the original data were higher than those for the same numbers of synergies extracted from the shuffled data. Furthermore, Figure 1 also represents the VAF across muscles (bottom).

We further checked that the muscle synergies extracted from the EMG data during walking accounted for the EMG data during running, or *vice versa*, to verify our assumption that both walking and running are achieved using the same muscle synergies. To this end, we separately extracted muscle synergies (*W*_walk_ and *W*_run_, respectively) from each of the EMG data matrix during walking (*EMG*_walk_) and running (*EMG*_run_) except a gait transition step, which consisted of 8 cycles × 200 time periods × 20 repetitions (5 repetitions and 15 repetitions; the changing and constant speed conditions, respectively) = 32000 time bins for each of the 22 muscles. The number of synergies was determined based on the same criteria as mentioned above. The weighting matrix, *W*_walk_, was held fixed in the NMF algorithm and activation coefficient matrix, *C*_run_, was updated to reconstruct *EMG*_run_ (Turpin et al., 2011). Then, we calculated the VAF, which indicated how the walking synergies, *W*_walk_, could explain *EMG*_run_. We applied this procedure to the opposite direction: the running synergies, *W*_run_, accounted for *EMG*_walk_.

### Quantifying the similarity of synergies across subjects

Functional sorting of the global synergies across each subject was initially performed by grouping muscle synergies based on the values of cosine similarity (*r* > 0.50) with that of an arbitrary reference subject using an iterative process. Subsequently, an averaged set of similar muscle synergies for all subjects was computed, and the similarity between the averaged muscle synergies and each synergy grouped across subjects was quantified (Torres-Oviedo and Ting, 2007; Hagio and Kouzaki, 2014).

### Statistics

The difference of the peak synergy activation phases, which indicated the maximum value within a gait cycle, between the changing and constant speed conditions was statistically analyzed using the Wilcoxon test. Statistics was performed within the similar synergies among all subjects across gait cycles.

## Results

The subjects walked or ran on a treadmill in the two different conditions to observe the different controlling characteristics at a gait transition. In the changing speed condition, as altering treadmill speed, the subjects spontaneously shifted their gait pattern between walking and running as the treadmill speed changed. Treadmill speed at the gait transition from walking to running was 6.63 ± 0.32 km/h, whereas subjects changed their gait from running to walking at 6.43 ± 0.90 km/h. In the constant speed condition, the treadmill speed was determined based on the averaged walk-to-run transition speed in each subject (Table 1). The subjects voluntarily and instantly changed their gait at the instruction from an experimenter.

**Table 1:** **Treadmill speed at the gait transition between walking and running across subjects**.

Transition type	Index	Subj.1	Subj.2	Subj.3	Subj.4	Subj.5	All subj.
Walk to run	avg [km/h]	6.64	6.48	6.84	6.60	6.60	6.63
	sd	0.18	0.13	0.51	0.32	0.32	0.32
Run to walk	avg [km/h]	6.55	7.13	6.68	6.33	5.47	6.43
	sd	0.12	0.21	0.31	0.74	1.49	0.90

*The average treadmill speed for 5 repetitions and their standard deviations at the walk-to-run and run-to-walk gait transition are shown. The transition speed was defined as the speed at the beginning of the first step with a flight phase in the walk-to-run transition and at the moment when the first step with a double stance phase began in the run-to-walk transition. Subj, subject*.

### Muscle activity

**Figure 2:**
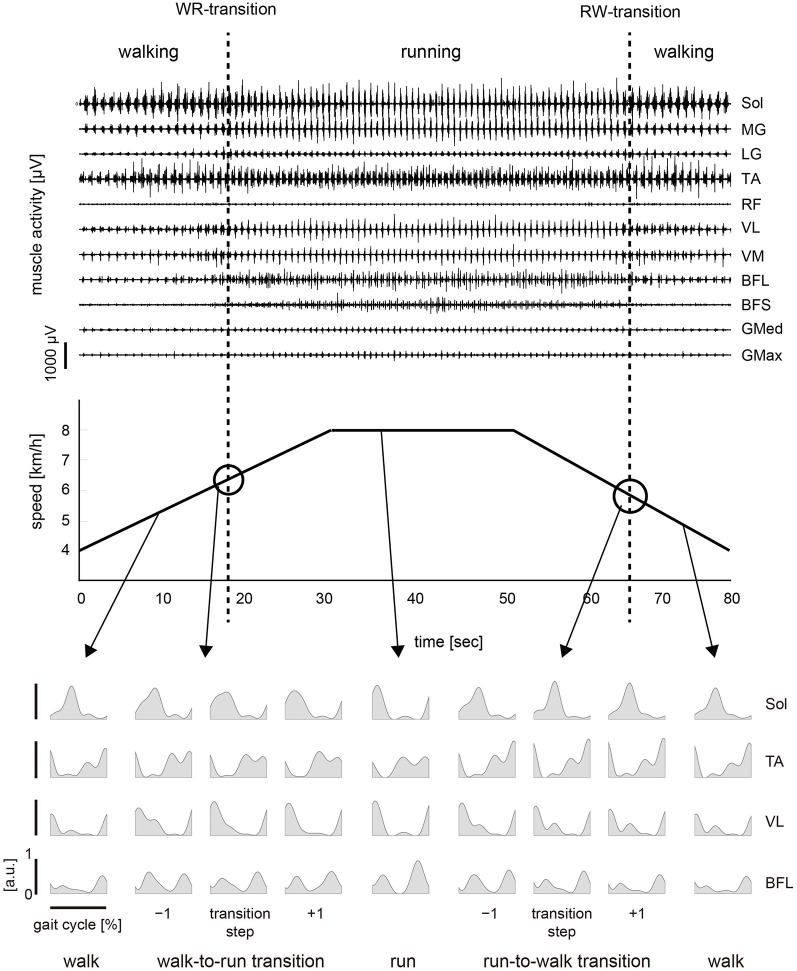
**Muscle activity and treadmill speed in the changing speed condition**. *Top*: Representative muscle activations across 11 right lower limb muscles in the changing speed condition. The activation traces were high-pass filtered at 100 Hz using a zero-phase-lag fourth-order Butterworth filter. Muscle names are indicated in an abbreviated form: Sol, soleus; MG, medial gastrocnemius; LG, lateral gastrocnemius; TA, tibialis anterior; RF, rectus femoris; VL, vastus lateralis; VM, vastus medialis; BFL, biceps femoris long head; BFS, biceps femoris short head; GMed, gluteus medius; GMax, gluteus maximus. *Middle*: The treadmill speed during the changing speed condition. The treadmill speed was first constantly accelerated. After the walk-to-run transition, the treadmill speed was maintained constant for 20 s, which was adequately faster than the walk-to-run transition speed. The speed was then constantly decelerated, and the run-to-walk transition occurred. *Bottom*: integrated electromyograms (iEMGs) of selected muscles [Sol, TA, VL and BFL] computed for single cycles during walking, running and −1 to +1 steps before and after the transition cycle. iEMGs were normalized with maximal value in all steps across each muscle and averaged for all repetitions. WR, walk-to-run. RW, run-to-walk.

**Figure 3:**
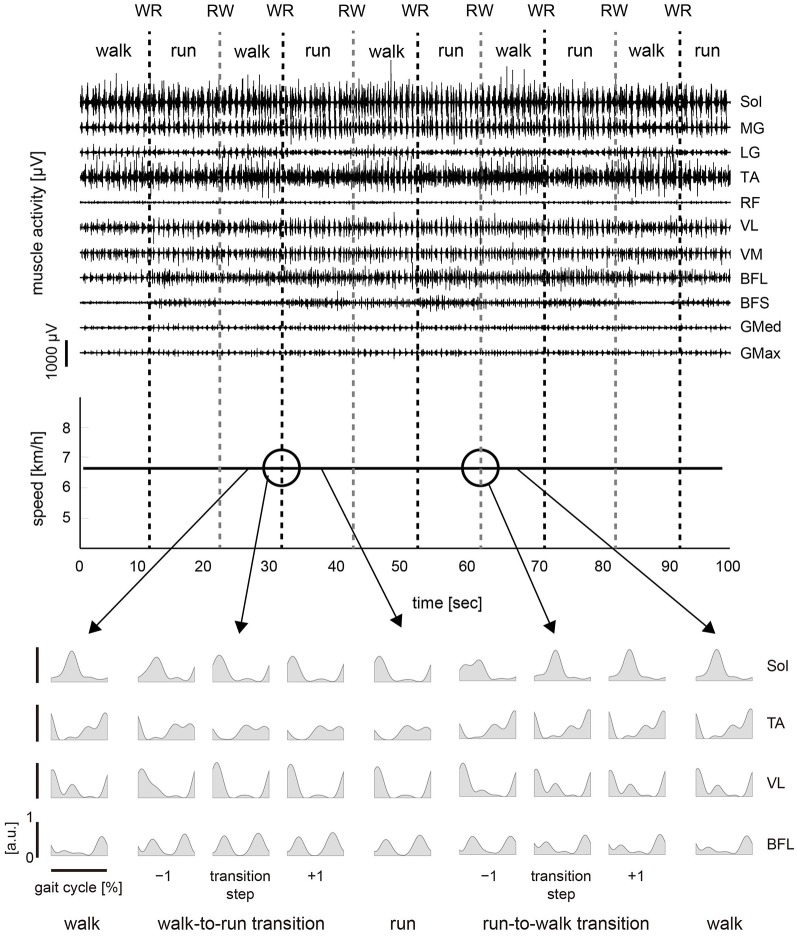
**Muscle activity and treadmill speed in the constant speed condition**. *Top*: Representative muscle activations across 11 right lower limb muscles during the constant speed condition. The activation traces were high-pass filtered at 100 Hz using a zero-phase-lag fourth-order Butterworth filter. *Middle*: The treadmill speed in the constant speed condition. The treadmill speed was constant and was determined as the average for the walk-to-run transition speeds in the five changing speed trials. Subjects were asked to walk or run for 10 s by turns following an experimenter’s instruction. One trial period was approximately 100 s. *Bottom*: iEMGs of selected muscles [Sol, TA, VL and BFL] computed for single cycles during walking, running and −1 to +1 steps before and after the transition cycle. iEMGs were normalized with maximal value in all steps across each muscle and averaged for all repetitions. WR, walk-to-run transition. RW, run-to-walk transition.

The activation pattern of each muscle in both conditions was consistent with that reported previously (Ivanenko et al., 2004; Cappellini et al., 2006) as observed in Figures 2, 3. In the changing speed condition, the intensity of proximal leg muscle (VL, VM BFL and BFS) activations gradually increased or decreased with the change of the treadmill speed during each gait pattern, whereas the intensity of each muscle activation was similar at the walk-to-run and the run-to-walk transition, respectively (Figure 2). Although the characteristics of muscle activation intensity observed in the constant speed condition were similar to those in the changing speed condition, the intensity of muscle activity was abruptly increased or decreased before and after gait transition (Figure 3).

### Muscle synergies

**Figure 4:**
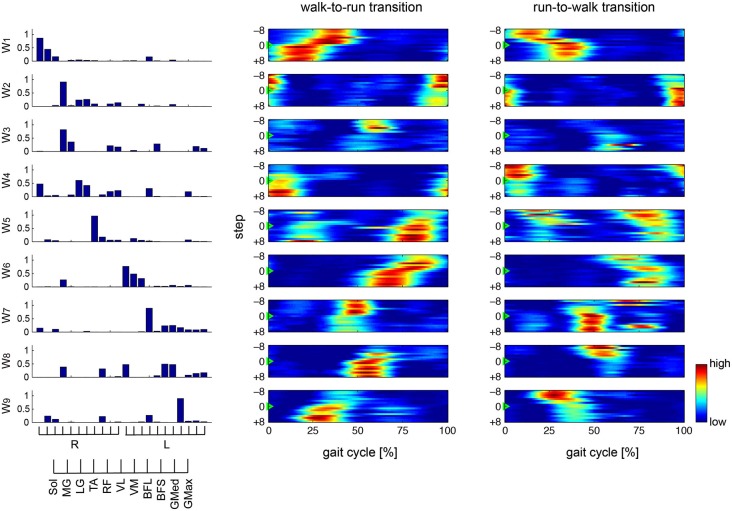
**Muscle synergies and activations in changing speed condition**. Muscle synergies and their activation profiles in a representative subject in the changing speed condition are shown. Bar graphs indicate muscle synergy vectors, i.e., weightings of each muscle within the synergy. Color map shows activation coefficients for each synergy across the walk-to-run and run-to-walk gait transitions. The vertical axis shows the gait transition step (defined as the 0th step ±8 gait cycles), and the horizontal axis indicates the phase of one gait cycle (from the onset of the right leg to the next; normalized to 200 time bins).

**Figure 5:**
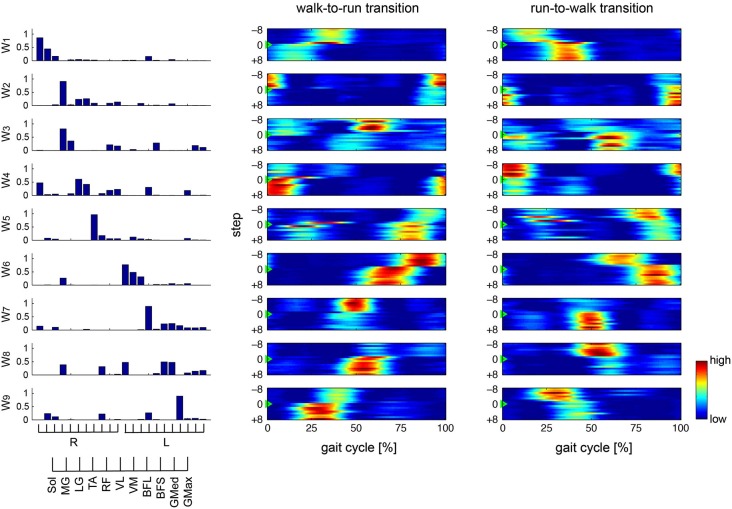
**Muscle synergies and activations in constant speed condition**. Muscle synergies and their activation profiles in a representative subject in the constant speed condition are shown. Bar graphs indicate muscle synergy vectors, i.e., weightings of each muscle within the synergy. Color map shows activation coefficients for each synergy across the walk-to-run and run-to-walk gait transitions. The vertical axis shows the gait transition step (defined as the 0th step ±8 gait cycles), and the horizontal axis indicates phase of one gait cycle (from the onset of the right leg to the next; normalized to 200 time bins).

To verify our methodological assumption that people modulate both walking and running through the same muscle synergies, we separately extracted 8.8 ± 1.30 and 8.4 ± 0.894 muscle synergies during walking (*W*_walk_) and running (*W*_run_), respectively. The synergies *W*_walk_ and *W*_run_ could adequately account for each of the EMG data during running and walking, i.e., *EMG*_run_ (VAF: 95.076 ± 0.536) and *EMG*_walk_ (VAF: 94.399 ± 0.894), respectively, which indicated that the muscle activations during both walking and running were achieved through the same muscle synergies. This result corresponded to our assumption for the extraction of muscle synergies (see Section Methods). Thus, we extracted 8.8 ± 1.10 muscle synergies from the total EMG data matrix in all of the trials. The muscle synergies and their activation profiles in a representative subject are shown in Figures 4, 5; in the changing and constant speed conditions, respectively. The bar graphs indicate the muscle synergy vectors, i.e., weightings of each muscle within the synergy. The color map shows the activation coefficients for each synergy. The vertical axis shows the gait transition step (defined as the 0th step) ± 8 gait cycles, and the horizontal axis indicates the phase of one cycle (from the onset of the right leg to the next; normalized to 200 time bins). In this subject, 9 synergies were extracted, and the structures and activation profiles were specific across the phase of the gait cycle. The synergies W_1_ and W_6_ were composed of the Sol, MG and LG of the right and left leg, respectively. The synergy W_1_ was activated in 30–40 % of the gait cycle during walking and in 10–25 % of the gait cycle during running. Near the gait transition, the peak activations of these synergies were shifted to the activation phase during the next gait pattern. The synergies W_2_ and W_7_, which were mainly composed of the TA of the right and left leg, respectively, were activated in the stance phase of the relevant leg. The intensity of these synergy activations decreased after the walk-to-run transition and increased after the run-to-walk transition. The synergy W_3_, which was composed o the TA and RF of the right leg, was recruited in the early swing phase during walking, but this synergy did not activate after the walk-to-run transition and before the run-to-walk transition, that is, during a running gait. The synergies W_4_ and W_8_ were dominant for the VM, VL and Sol of the right and left leg, respectively. These synergies activated in the stance phase of each leg just before the walk-to-run transition and after run-to-walk transition. The synergies W_5_ and W_9_ predominantly consisted of the BFL of the right and left leg, respectively. The peak activations of these synergies were approximately 40% of the gait cycle during walking and shifted to 30% of the gait cycle during running. The intensity of the activations during running was greater than that during walking. For other subjects, the intensity of the principal synergy activation was altered in a similar fashion before and after the gait transition, whereas the consistent phase shift of the synergy activations was observed only in the synergies, which were composed of the Sol, MG and LG (W_1_ and W_6_ in the case of the representative subject). The characteristic activation patterns of all synergies during walking and running and during a gait transition were similar between the changing and constant speed conditions. The activation profiles before and after gait transition, however, were different between the two conditions. In the changing speed condition, the peak activation phase was gradually shifted from the walking phase to the running phase near a gait transition (Figure 4), while an abrupt change was observed during the constant speed condition (Figure 5).

**Figure 6:**
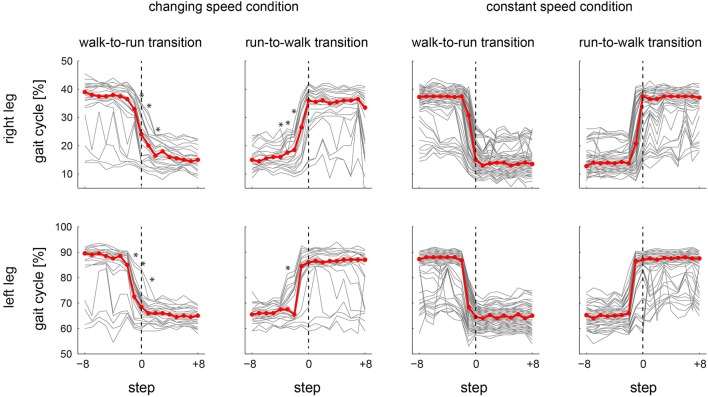
**Peak activation phase in the specific synergies**. The peak synergy activation phases, which were composed of the Sol, MG and LG in the right (top) and left (bottom) lower limb, respectively, in the gait transition step (defined as the 0th step ±8 steps) for all subjects are shown. The gray lines indicate the individual trials for all subjects and repetitions. The red lines are the median value across gait steps. The asterisk symbol above the steps in the changing speed condition indicated the significant difference of the activation phases in between changing and constant speed conditions (*p* < 0.05). See text for detail.

We focused on the synergies that were composed of the Sol, MG and LG (W_1_ and W_6_ in the case of the representative subject) and that were common among all subjects, to compare the two different controlling strategies between the changing and constant speed conditions (Figure 6). Figure 6 shows the phase of the peak synergy activations in the gait transition step ± 8 steps for all subjects across conditions. We counted which to which steps were influenced by a gait transition before and after a gait transition step to compare the difference of the activation phase shift in between changing and constant speed conditions. The initial and last steps influenced by a gait transition were defined as the first and last steps, which exceeded 2 SD (standard deviation) of −8 to −5 steps before the transition step and of 5 to 8 steps after the transition step, respectively. As a result, in the synergy W_1_, 6 and 2 steps were influenced by a gait transition in the changing speed condition during walk-to-run and run-to-walk transition, respectively, whereas 1 and 3 steps were influenced in the constant speed condition walk-to-run and run-to-walk transition, respectively. In the case of the synergy W_6_, 8 steps were influenced in the changing speed condition during both walk-to-run and run-to-walk transitions, whereas 1 and 4 steps were influenced in the constant speed condition during walk-to-run and run-to-walk transition, respectively. Although the influenced steps in the synergy W_1_ in the changing speed condition during run-to-walk transition were fewer than in the constant speed condition, the influenced steps before the gait transition step were started earlier (3 steps before) than in the constant speed condition (in W_1_, 1 steps before during both walk-to-run and run-to-walk transition; and in W_6_, 2 steps before during both walk-to-run and run-to-walk transition). Accordingly, during the walk-to-run condition in the changing speed condition, the activation phase gradually shifted before and after the gait transition. In the case of the constant speed condition, the activation phases were shifted at 1 step before the gait transition and were stable in the next gait phase at a gait transition step. Especially, at 0 to +3 steps (right leg; Figure 6 top) and −1 to +1 steps (left leg; Figure 6 bottom), the significant differences of the phase of the peak synergy activation were observed between the changing and constant speed condition s (*p* < 0.05). Furthermore, during the run-to-walk transition, the synergy activation phases were gradually shifted before the gait transition in the changing speed condition, whereas abrupt changes were observed in the constant speed condition. During the run-to-walk transition, the phases of the peak synergy activations were significantly different between the two conditions at −4 to −2 steps (right leg; Figure 6 top) and −3 step (left leg; Figure 6 bottom) (*p* < 0.05).

**Figure 7:**
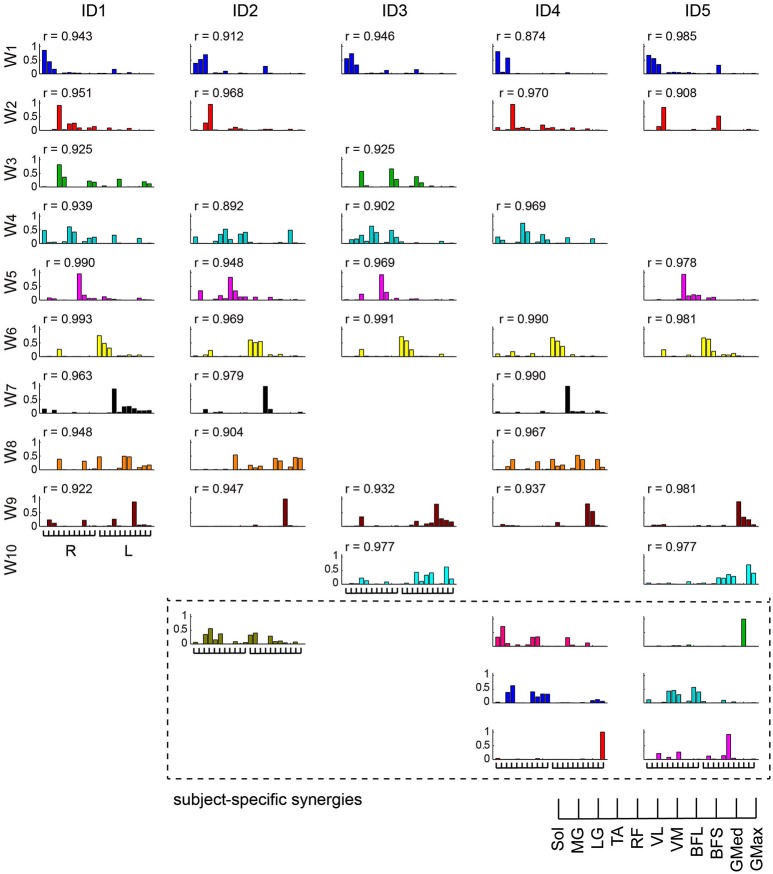
**Muscle synergies across subjects**. Muscle synergies were grouped by their similarity and sorted across subjects. The similar muscle synergies across subjects are shown in the same color. The synergies surrounded by dashed lines are subject-specific. The *r*-value represents the cosine similarities between the averaged muscle synergies from the initial sorting, and each original synergy is grouped across subjects (see Methods section).

We categorized similar muscle synergies into groups and sorted them across subjects (Figure 7). Synergies W_1,6_, which were composed of the Sol, MG and LG in the right and left legs, and W_9_, which was dominant for the left BFL, were common for all subjects (0.874 < *r* < 0.993). Furthermore, in 4 subjects, synergies W_2,4,5_ were similar (0.892 < *r* < 0.990), while synergies W_3,7,8,10_ were common in two or three subjects (0.904 < *r* < 0.979). However, the other synergies (surrounded with dashed lines in Figure 7) were subject-specific.

## Discussion

The purpose of this study was to identify the neural mechanisms of gait transition between walking and running based on muscle synergies. We observed gait transition using two different tasks. The main finding was that gait transition was low-dimensionally controlled by muscle synergies, which were also recruited during walking and running gaits, and was achieved by altering the activation profile of a few specific muscle synergies. Furthermore, a gradual shift of the synergy activations before and after the gait transition was observed during the changing speed condition, whereas the constant speed condition required one or two steps to change the gait patterns.

### Spontaneous gait transition

In the changing speed condition, we constantly accelerated or decelerated the treadmill speed to provoke a spontaneous gait transition between walking and running. As a result, the activation phases of the specific synergies (W_1_ and W_6_ shown in Figure 4), which were dominant for the Sol, MG and LG in the right and left legs, respectively, were gradually altered before and after the gait transition (Figure 6 in the changing speed condition). These synergies were activated near the last stance phase of the relevant leg during walking, and the synergy activation phase was altered to an earlier phase of the gait cycle during running. Near a gait transition, the activation phases shifted from those during walking to during running, and the alteration was quite gradual. Neurophysiologically, our result indicates that the spontaneous gait transition was not abrupt but gradually occurred due to the prepared events. This gradual alteration near a gait transition is supported by previous evidence that gait transitions are not abrupt events, although several researchers (Hreljac, 1995; Diedrich and Warren, 1998; Abernethy et al., 2002; Raynor et al., 2002) have assumed that gait transitions are abrupt events. For humans, Li and Hamill (2002) reported differences in the ground reaction forces of the steps leading up to the walk-to-run gait transition, suggesting that this transition occurs gradually. Segers et al. (2006) observed differences in the spatiotemporal characteristics in the steps leading to both the walk-to-run and run-to-walk transitions. Our findings suggest that the gradual shift of the synergy activation phase relate to these previously observed kinematical factors. The CNS might receive information to trigger a gait transition and prepare for the changing gait pattern by gradually shifting the activation timing of the specific synergies.

Here, our question is why the CNS changes the synergy activations, or what constitutes the trigger of a gait transition. In the concept of locomotor central pattern generators, a gait transition was supposed to be occurred by the increment of the stimulation to a specific region in the brain stem called Mesencephalic Locomotor Region (MLR; Shik et al., 1966; Ijspeert, 2008). Hence, the trigger of a gait transition will be something increasing the stimulation to MLR, which lead to the change of activation of the specific muscle synergies (W_1_ and W_6_ shown in Figure 4). Many researchers have focused on this problem and suggested some afferent information as the trigger of a gait transition. Hreljac (1995) showed that the velocity of ankle flexion was the critical factor, suggesting that the dorsiflexor muscles overexerted a dorsiflexion torque in the initial swing phase near a gait transition and therefore served as a determinant for the walk-to-run transition. These results were supported by the later studies (Prilutsky and Gregor, 2001; Bartlett and Kram, 2008) and our study also had similar findings. The synergy activation levels, which were dominant for the TA and recruited during the swing phase, were decreased after the walk-to-run gait transition (Figure 4, W_3_). Other researchers concluded that the walk-to-run transition in humans might be triggered by reaching a critical angle between the thighs (Minetti et al., 1994) or that the force generating capability of the plantar flexor muscles becomes greatly impaired as the walking speed approaches the characteristic speed of a gait transition (Neptune and Sasaki, 2005). These factors, however, cannot completely explain the run-to-walk gait transition. The difference between walk-to-run and run-to-walk transitions was previously supposed by the different neurophysiological triggers: the walk-to-run transition might be triggered by the increased sense of effort due to the higher demand of swing-related activation of the TA, RF and BFL whereas the run-to-walk transition might be triggered by the sense of effort due to the higher support-related activation of the Sol, MG and VM (Prilutsky and Gregor, 2001). Furthermore, the kinematical difference between the walk-to-run and run-to-walk gait transitions has been discussed previously, but the controlling mechanisms remain unclear (Segers et al., 2006). In our study, despite the similar gait transition speed (Table 1), the phase shift of the synergy activations showed different patterns between the walk-to-run and run-to-walk transitions. In the walk-to-run transition, the gradually changing synergy activations were continued after the transition, whereas the synergy activation profiles were nearly stabilized upon the transition in the run-to-walk transition (Figure 6). Thus, we suggest that the walk-to-run and run-to-walk gait transitions were controlled by different neural mechanisms through muscle synergies. Accordingly, these different triggers might lead to these two gait transitions.

Despite some evidence, the trigger of a gait transition remains unclear. However, the gradual regulation of the synergy activation phases demonstrated that the CNS responded to some informative factors near a gait transition. Therefore, our result that the activations of the muscle synergies were gradually changed near a gait transition suggests that the CNS may receive afferent information, which represents the trigger of a gait transition, and gradually regulates the descending neural input to the synergies.

### Voluntary gait transition

In this study, we assumed two different types of a gait transition: not only a spontaneously occurring transition through gradually changing the gait speed (an experimental protocol that has been previously well-studied (Thorstensson and Roberthson, 1987; Li, 2000; Li and Hamill, 2002; Segers et al., 2006)) but also a voluntarily performed transition (regardless of gait speed). Because subjects voluntarily changed their gait pattern in the constant speed condition, the neural control strategy was considered to be different from the spontaneous gait transition in the changing speed condition. Indeed, the activation phases were shifted at 1 step before the gait transition and stable during the next gait phase at a gait transition step (Figure 5) although the changing speed condition required several steps to gradually change the synergy activations (Figure 4). In this condition, the trigger of a gait transition was the instruction from an experimenter. Therefore, the CNS induced a gait transition by changing only the descending neural input to the specific muscle synergies without afferent information. A previous study reported different adaptations between walking and running, indicating that their gait patterns have fundamentally different neural control mechanisms (Ogawa et al., 2012). Therefore, the two gait patterns must be controlled and switched at a higher level than the neural pathway of muscle synergies because the walking and running gaits were achieved by similar muscle synergies. The CNS shifts the synergy activation phases by switching the neural pathways and changing the gait patterns between walking and running.

In summary, the CNS low-dimensionally controls the gait transition between walking and running by regulating the activation profiles of specific muscle synergies. Furthermore, a spontaneous gait transition, near which gradual shifts of the synergy activation phases were observed, might occur based on afferent information, whereas a voluntary gait transition can be achieved by changing only the descending neural input to the muscle synergies without afferent information within one or two steps.

## Author contributions

Conception and design of the experiments: Shota Hagio, Mizuho Fukuda, Motoki Kouzaki. Collection, analysis and interpretation of data: Shota Hagio, Mizuho Fukuda. Drafting the article or revising it critically for important intellectual content: Shota Hagio, Mizuho Fukuda, Motoki Kouzaki. Final approval of the version to be published: Shota Hagio, Mizuho Fukuda, Motoki Kouzaki.

## Conflict of interest statement

The authors declare that the research was conducted in the absence of any commercial or financial relationships that could be construed as a potential conflict of interest.
